# A phase 2 study to assess the pharmacokinetics and pharmacodynamics of CPX-351 and its effects on cardiac repolarization in patients with acute leukemias

**DOI:** 10.1007/s00280-019-03856-9

**Published:** 2019-05-16

**Authors:** Tara L. Lin, Laura F. Newell, Robert K. Stuart, Laura C. Michaelis, Eric Rubenstein, Helen S. Pentikis, Timothy Callahan, Donna Alvarez, Barry D. Liboiron, Lawrence D. Mayer, Qi Wang, Kamalika Banerjee, Arthur C. Louie

**Affiliations:** 1 University of Kansas Medical Center, 3901 Rainbow Blvd., Kansas City, KS USA; 2 Knight Cancer Institute, Oregon Health and Science University, Portland, OR USA; 3 Hollings Cancer Center, Medical University of South Carolina, Charleston, SC USA; 4 Medical College of Wisconsin, Milwaukee, WI USA; 5 Oncology and Hematology Specialists, Franciscan Health, Indianapolis, IN USA; 6 SAJE Consulting, Baltimore, MD USA; 7 Biomedical Systems, St. Louis, MO USA; 8 Celator/Jazz Pharmaceuticals, Palo Alto, CA USA

**Keywords:** Acute myeloid leukemia, Acute lymphoblastic leukemia, Cardiac repolarization, Pharmacokinetics, Pharmacodynamics

## Abstract

**Purpose:**

Daunorubicin can induce left ventricular dysfunction and QT interval prolongation. This study assessed the effects of CPX-351, a liposomal encapsulation of cytarabine and daunorubicin, on cardiac repolarization.

**Methods:**

Twenty-six adults with acute leukemia were treated with CPX-351 for 1–2 induction cycles and ≤ 4 consolidation cycles. The primary endpoint was mean change in QTcF from baseline.

**Results:**

Mean QTcF changes were < 10 ms at all time points. No clinically meaningful effects on heart rate, QRS interval, PR interval, or QTcB were observed. Estimated mean half-lives for total cytarabine and daunorubicin were > 30 h. Thirteen (50%) patients achieved remission. The most common adverse events were febrile neutropenia, fatigue, and nausea.

**Conclusions:**

The cytarabine and daunorubicin in CPX-351 liposomes were metabolized and excreted similarly to conventional formulation; however, plasma pharmacokinetics were altered. CPX-351 did not prolong the QT interval, suggesting that CPX-351 may induce less cardiotoxicity than previously reported for conventional daunorubicin.

**Trial registration:**

Clinicaltrials.gov identifier: NCT02238925.

**Electronic supplementary material:**

The online version of this article (10.1007/s00280-019-03856-9) contains supplementary material, which is available to authorized users.

## Introduction

The combination of cytarabine plus an anthracycline, such as daunorubicin (7 + 3 regimen), has been a standard of care for acute myeloid leukemia (AML) induction for decades [1]. Outcomes among patients aged > 65 years are poor, and 5-year survival rates remain in the single digits. Because the median age at diagnosis of AML is 68 years, most patients face an unfavorable prognosis [2, 3]. More effective treatment strategies are needed for patients with particularly poor outcomes, such as older patients or patients with secondary leukemia [4, 5]*.*

In vitro experiments have demonstrated that a 5:1 molar ratio of cytarabine to daunorubicin is synergistic, yielding maximum antileukemia activity compared with the other drug ratios examined; depending on the cell line studied, other ratios ranging from 1:1 to 1:10 could be antagonistic [6]. Since cytarabine and daunorubicin pharmacokinetics differ [7, 8], their molar ratio may vary substantially over time in vivo when administered as conventional free drugs [9].

CPX-351 (Vyxeos®; Jazz Pharmaceuticals, Inc.), a liposomal encapsulation of cytarabine and daunorubicin at a 5:1 synergistic molar ratio [6], was developed using the CombiPlex® platform (Jazz Pharmacueticals, Inc.), which employs nanoscale carriers to maintain agents with different pharmacokinetic profiles at a synergistic ratio [10]. In a phase 1 study, CPX-351 maintained a synergistic cytarabine:daunorubicin ratio in plasma for up to 24 h after intravenous injection [11]. In the pivotal phase 3 study of older patients with newly diagnosed, high-risk/secondary AML, CPX-351 significantly prolonged median overall survival versus 7 + 3 (9.56 vs 5.95 months, respectively; HR = 0.69; 1-sided *P* = 0.003). In addition, CPX-351 was associated with a significantly higher overall remission rate (complete remission [CR] + CR with incomplete recovery of platelets or neutrophils [CRi]: 47.7% vs 33.3%; 2-sided *P* = 0.016) [12]. The safety profile of CPX-351 was generally consistent with that of 7 + 3. These data led to approval of CPX-351 by the US Food and Drug Administration and the European Medicines Agency for the treatment of adults with newly diagnosed therapy-related AML or AML with myelodysplasia-related changes [13]. Preliminary data suggest that CPX-351 may also be active against other hematological malignancies (eg, acute lymphoblastic leukemia [ALL]) and warrants further investigation [11, 14].

A number of anticancer drugs have negative effects on cardiovascular health, including prolongation of cardiac repolarization, which can lead to fatal ventricular arrhythmia [15]. QT interval, the time between ventricular depolarization and subsequent repolarization, serves as a surrogate marker for risk for arrhythmia [16]. Anthracyclines, including daunorubicin, can induce acute and chronic cardiotoxicity, such as QT interval prolongation and left ventricular dysfunction [15, 17–20]. In some cases, anthracycline-induced cardiac damage leads to heart failure, especially among patients with a high cumulative anthracycline dose [15, 18]. Consequently, the conventional 7 + 3 regimen has historically been restricted to patients with adequate cardiac function, and the cumulative anthracycline dose is usually limited to ≤ 450 mg/m^2^ [18]. Clinical studies evaluating left ventricular ejection fraction (LVEF), endomyocardial biopsies, and the incidence of congestive heart failure suggest that liposomal encapsulation of single-agent daunorubicin reduces cardiotoxicity versus conventional daunorubicin [18, 21, 22]. Thus, in addition to its benefit in prolonging survival and remission rates compared with conventional 7 + 3, CPX-351 might also attenuate the risk of potentially fatal anthracycline-induced cardiotoxicity. The International Council on Harmonisation guidelines recommend evaluating the effects of new therapies on the QT interval [23]. Therefore, this phase 2 study was designed to assess the effects of CPX-351 on cardiac repolarization and further characterize the CPX-351 pharmacokinetic profile in patients with acute leukemia.

## Patients and methods

### Study design

This open-label, single-arm, phase 2 study of the pharmacokinetics, pharmacodynamics, and cardiac safety of CPX-351 (ClinicalTrials.gov identifier: NCT02238925) enrolled patients from 5 sites within the United States. This study was conducted according to the provisions of the Declaration of Helsinki and the International Conference on Harmonisation–Good Clinical Practice. The study protocol was approved by the institutional review board at each study site, and all patients provided written informed consent before enrollment.

### Patient populations

Patients had newly diagnosed or relapsed/refractory AML, relapsed/refractory ALL, or myelodysplastic syndrome with an International Prognostic Scoring System score ≥ 1.5. Eligible patients were aged ≥ 18 to ≤ 80 years, had an Eastern Cooperative Oncology Group performance status ≤ 2, and had a life expectancy of ≥ 3 months. Additional key eligibility criteria included cardiac ejection fraction ≥ 50% by echocardiography or multigated acquisition scan and screening and baseline Fridericia's corrected QT interval (QTcF) < 470 ms.

Healthy volunteers and patients taking medications known to prolong the QT interval are generally excluded from QT interval prolongation studies, but in this study a small number of these medications were considered important for patient supportive care. In these patients, eligibility was assessed at baseline with a screening electrocardiogram while taking the potentially problematic medication. If the QT interval was normal at baseline, these patients were enrolled and the necessary medications were administered. Patients were not enrolled if they had significant cardiac abnormalities, including rhythm abnormalities, unexplained syncope, atrioventricular or bundle branch blockage, abnormal T or U wave morphology, or other evidence of myocardial impairment resulting in heart failure per the New York Heart Association criteria (Class III or IV staging). Patients with prior cumulative anthracycline exposure > 368 mg/m^2^ (Online Resource Table S1) were excluded. Patients with a history of Wilson disease or other copper-related metabolic disorders were excluded.

### Study treatment

First induction consisted of 100 units/m^2^ CPX-351 (100 mg/m^2^ cytarabine and 44 mg/m^2^ daunorubicin) via 90-min intravenous infusion on Days 1, 3, and 5. Patients who did not respond to first induction could receive a second induction of 100 units/m^2^ CPX-351 on Days 1 and 3. Patients in CR/CRi could receive ≤ 4 consolidation cycles of 65 units/m^2^ CPX-351 (65 mg/m^2^ cytarabine and 29 mg/m^2^ daunorubicin) on Days 1 and 3. The 60-day follow-up phase began after completion of the treatment phase.

### Pharmacodynamics (QT assessment)

Patients were evenly divided into 2 groups for pharmacokinetic evaluations (Groups A and B; see methods for *Pharmacokinetics*). In both groups, triplicate 12-lead electrocardiograms were extracted from Holter recordings on Days 0, 1, and 5 at pre-dose and at the following time points after the start of drug infusion (SOI): 0.75, 1.5, 2, 3, 4, 6, 8, 12, and 24 h, and additionally on Day 5 at 26, 28, 32, and 36 h post-SOI. Single 12-lead electrocardiograms were collected on Days 8, 9, 12, 14, 19, 21, 60, and either at end-of-study (EOS) or early termination. Computer-Assisted Measurement of Intervals software was used to annotate the Global Superimposed Median Beat. The QT interval was measured from the earliest detection of depolarization in any lead (beginning of Q or R wave) to the latest detection of repolarization in any lead (end of T wave). The primary endpoint for cardiac repolarization assessment was the mean change from the time-matched baseline of QTcF following the first induction. Following examination of electrophysiologic data, a concentration-QTc analysis was performed for all observations.

### Pharmacokinetics

Plasma pharmacokinetic samples were collected from all patients on Days 1 and 5 of the first induction at pre-dose and at the following times post-SOI: 45 and 90 min and 2, 4, 6, 8, and 24 h, and additionally on Day 5 at 28, 48, 72, 96, 168, 336, and 384 h post-SOI. In Group A, pharmacokinetic samples were processed to determine levels of total cytarabine and daunorubicin, as well as their metabolites (Ara-U and daunorubicinol). In Group B, samples were processed to assess plasma concentrations of total and unencapsulated (ie, “free”) cytarabine and daunorubicin and their metabolites; plasma concentrations of encapsulated cytarabine and daunorubicin were to be calculated by subtracting free from total concentrations. In addition, 24-h urine samples were obtained from a subset of 6 patients from Group B for 5 days (0–24, 24–48, 48–72, 72–96, and 96–120 h) following the end of first induction.

Plasma and urine concentrations of daunorubicin, cytarabine, and their respective metabolites were measured by validated liquid chromatography and mass-spectrometry (LC/MS/MS) methods. Briefly, plasma samples were acidified using 5% acetic acid in acetonitrile, which ruptured the liposomes and released encapsulated cytarabine and daunorubicin. After acidification, samples were centrifuged at 3000 rpm, and the supernatants were collected and dried at 40 °C. Samples were reconstituted in 0.5% acetic acid in water, separated using LC, and detected using MS. Urine samples were treated with methanol:water (25:75) and centrifuged at 3000 rpm. The supernatant was evaporated to dryness and reconstituted in water. Reconstituted samples were analyzed for cytarabine, daunorubicin, and metabolites using LC/MS/MS. The lower limit of quantification for daunorubicin and daunorubicinol was 5 and 3.83 ng/mL, respectively, and for cytarabine and Ara-U was 250 ng/mL in urine.

In both groups, serum copper sampling was performed at Days 1, 5, 9, 14, 19, and 21 of the first induction, prior to every course of treatment, at end of treatment, and 60 ± 10 days after EOS.

Plasma and urine pharmacokinetic parameters were calculated by non-compartmental pharmacokinetic analysis using Phoenix WinNonlin 6.3 (Pharsight, St. Louis, MO, USA). Data management and generation of the pharmacokinetic input files were performed using R version 3.0.3 (R Foundation for Statistical Computing, Vienna, Austria). Following Day 5 administration of CPX-351, steady-state plasma pharmacokinetic parameters were reported, including maximum observed concentration (*C*_max_), time of *C*_max_, area under the curve from 0 to 48 h following CPX-351 administration (AUC_0–48 h_), total body clearance (calculated as dose/AUC_0–48 h_), volume of distribution based on the terminal elimination phase, terminal-phase half-life (*t*_1/2_), and metabolite-to-parent ratio (calculated using AUC values after CPX-351 administration on Day 5). Urine pharmacokinetic parameters were also reported, including percent of analyte eliminated in urine for 48 h after CPX-351 administration on Day 5 (Ae) and renal clearance (calculated as dose × Ae/AUC_0–48 h_).

The scatter plot for change in the corrected QT interval (QTc) versus plasma concentrations was presented graphically to assess the linear relationship. If the relationship appeared to be non-linear based on the scatter plots and R2, a non-linear maximum effect model was explored. The concentration–response relationship between plasma cytarabine and daunorubicin levels and electrocardiogram/Holter-assessed QT interval data was examined using linear mixed-effects modeling. Three linear models were applied to the pharmacokinetics/QTc analysis data set: (1) linear model with an intercept; (2) linear model with mean intercept fixed to 0 with variability; and (3) linear model with mean intercept fixed to 0 without variability. Time-matched plasma concentrations were included as covariates, when applicable.

### Efficacy and safety evaluation

In general, the AML patient’s response to induction therapy was determined on the first day when all criteria for CR/CRi or treatment failure were met, with recovery of counts assessed within 14 days of the recovery. Patients with AML and ALL were assessed for response according to the 2010 European LeukemiaNet criteria and the 2013 National Comprehensive Cancer Network criteria, respectively [24, 25].

Adverse events (AEs) were coded using the medical dictionary for regulatory activities (version 16.0). Any LVEF < 50% was recorded as an AE, and any decrease in LVEF > 10% resulting in a nadir LVEF < 50% was reported as a serious AE.

### Statistical analyses

A sample size of ≥ 24 patients was enrolled and treated to enable detection of a mean change from baseline QTcF associated with a 1-sided upper 95% confidence interval (CI) width of < 5 ms, assuming a standard deviation (SD) of 8 ms for the QTcF change from baseline and ≤ 2 of the pharmacokinetic time points were likely to demonstrate a change of ≥ 5 ms.

Safety and efficacy analysis populations were composed of all 26 patients who received ≥ 1 dose of CPX-351. The pharmacokinetic and pharmacodynamic analysis population included 26 patients from whom pharmacokinetic and QT data were collected. Descriptive statistics were generated at each electrocardiogram extraction time, based on mean change from the time-matched baseline in QTcF, QT/QTc, heart rate, PR interval, and QRS interval; corresponding 90% 2-sided CIs were calculated. Repeated-measures regression analyses were performed to explore the relationship between mean changes in QTcF and plasma concentrations of cytarabine, daunorubicin, and their metabolites. Data manipulation was performed with SAS (version 9.2; SAS Institute, Inc.).

## Results

### Patient population

Twenty-six patients were enrolled and treated with CPX-351. Patient disposition is shown in Online Resource Table S2. The median age was 67 years, and 54% of patients were male. Twenty-four (92%) patients had AML and 2 (8%) had relapsed/refractory ALL. Other patient demographics are shown in Table 1.

**Table 1:** Summary of baseline demographics and characteristics

Characteristic	*N* = 26
Median (range) age, year	67.0 (37–80)
< 60 year, *n* (%)	6 (23)
≥ 60 year, *n* (%)	20 (77)
Sex, *n* (%)	
Male	14 (54)
Female	12 (46)
Race, *n* (%)	
White	25 (96)
Black or African American	1 (4)
ECOG PS, *n* (%)	
0	7 (27)
1	16 (62)
2	3 (12)
Disease type, *n* (%)	
Newly diagnosed de novo AML	7 (27)
Newly diagnosed secondary AML	6 (23)
Relapsed/refractory AML	11 (42)
Relapsed/refractory ALL	2 (8)
Prior anthracycline exposure, *n* (%)	14 (54)
Daunorubicin	6 (23)
Idarubicin	6 (23)
Mitoxantrone	2 (8)
Doxorubicin	1 (4)
Epirubicin	1 (4)
Prior therapy type,^a ^*n* (%)	
Radiation therapy	7/20 (35)
Chemotherapy	17/20 (85)
Hypomethylating agent	3/20 (15)
Other	8/20 (40)
Baseline renal function (CrCl), *n* (%)	
Normal ( ≥ 90 mL/min)	12 (46)
Mild impairment (60–89 mL/min)	8 (31)
Moderate impairment (30–59 mL/min)	6 (23)
Severe impairment (15–29 mL/min)	0 (0)

*ECOG PS* Eastern Cooperative Oncology Group performance status, *AML* acute myeloid leukemia, *ALL* acute lymphoblastic leukemia, *CrCl* creatinine clearance

^a^Patients with ≥ 1 occurrence within a therapy type are counted once for that therapy type. Patients with ≥ 1 type of prior therapy are counted once for each therapy type

### CPX-351 exposure

All patients received ≥ 1 induction cycle of CPX-351; 3 (12%) patients received a second induction cycle. Three (12%), 2 (8%), and 1 (4%) patients received 1, 2, and 3 consolidation cycles of CPX-351, respectively. The median time on study was 92 days; patients received a mean (SD) of 3.7 (1.5) total infusions and 696 (254) units CPX-351, equivalent to 696 mg cytarabine and 306 mg daunorubicin.

### Effect of CPX-351 on QTc

**Fig. 1:**
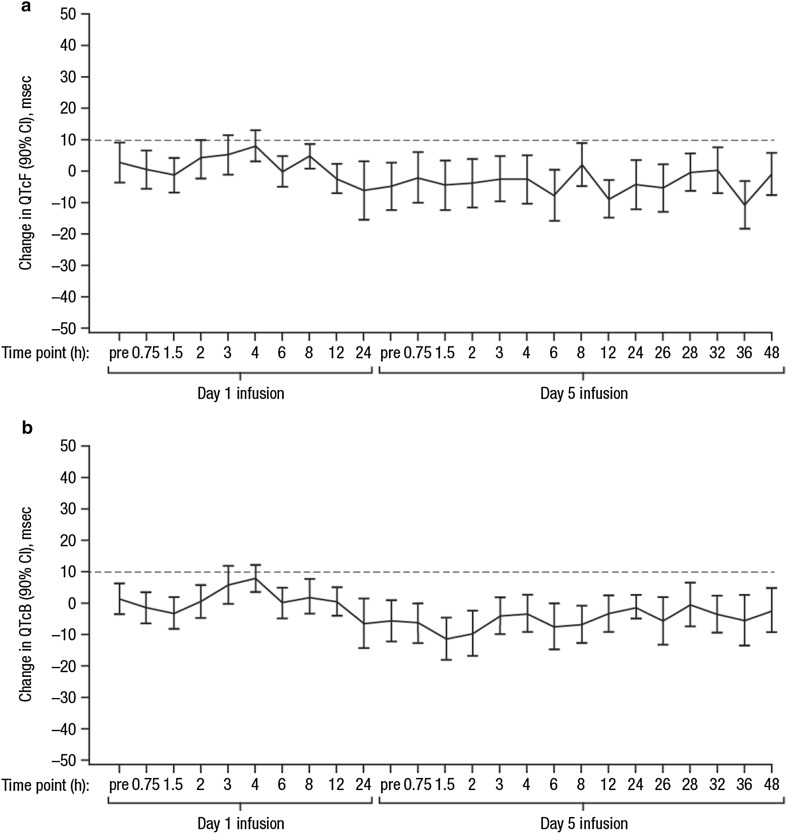
Mean change from baseline in QTcF (**a**) and QTcB (**b**). Mean values are shown with their 95% CIs. Mean changes in QTcF were < 10 ms at all time points and upper limits of the 2-sided 90% CI exceeded 10 ms at only 2 time points (Day 1 at 3 h post-SOI: 10.7 ms [mean = 5.3]; Day 1 at 4 h post-SOI: 12.3 ms [mean = 8.0]). *QTcF* Fridericia's corrected QT interval, *CI* confidence interval, *QTcB* Bazett's corrected QT interval, *SOI* start of infusion

No clinically meaningful changes in QTcF were observed during the intensive QT monitoring period using a Holter instrument (Fig. 1). Mean changes in QTcF were < 10 ms at all time points, and upper limits of the 2-sided 90% CI exceeded 10 ms at only 2 time points (Day 1 at 3 h post-SOI: 10.7 ms [mean = 5.3]; Day 1 at 4 h post-SOI: 12.3 ms [mean = 8.0]); these changes were not statistically significant. No patient had a change in QTcF ≥ 60 ms from baseline, and no QTcF value was > 500 ms.

Similarly, no clinically important effect of CPX-351 was observed on heart rate, QRS interval, or PR interval. No patient had a > 20% reduction in heart rate resulting in a heart rate < 50 bpm; 7 patients had a > 20% increase in heart rate resulting in a heart rate > 100 bpm. No patient had a QRS interval duration > 120 or < 60 ms, and changes in the duration of QRS were < 20% from baseline. No patient had a PR interval > 200 or < 120 ms, and changes in PR intervals were < 20% from baseline.

Linear mixed-effects modeling based on pharmacokinetic and QTcF data were used to test the difference of both the slope and intercept terms from 0. The analysis showed that none of the slope or intercept estimates were significantly different from 0, indicating no concentration effects on QTcF due to components or metabolites of CPX-351 (Table 2).

**Table 2:** Regression results from concentration–response analysis

Metabolite	Parameter	Estimate	*P* value
QTcF			
Total Ara-U	Intercept (msec)	5.44	0.7785
	Slope (ln(msec)/ln(ngmL))	− 1.20	0.7115
Total cytarabine	Intercept (msec)	1.51	0.9484
	Slope (ln(msec)/ln(ngmL))	− 0.25	0.9132
Total daunorubicin	Intercept (msec)	− 9.74	0.4963
	Slope (ln(msec)/ln(ngmL))	0.93	0.5377
Total daunorubicinol	Intercept (msec)	8.19	0.3290
	Slope (ln(msec)/ln(ngmL))	− 2.25	0.2823
QTcB			
Total Ara-U	Intercept (msec)	16.24	0.3612
	Slope (ln(msec)/ln(ngmL))	− 3.11	0.2752
Total cytarabine	Intercept (msec)	41.89	0.1402
	Slope (ln(msec)/ln(ngmL))	− 4.28	0.1046
Total daunorubicin	Intercept (msec)	11.13	0.5559
	Slope (ln(msec)/ln(ngmL))	− 1.51	0.4258
Total daunorubicinol	Intercept (msec)	6.63	0.3521
	Slope (ln(msec)/ln(ngmL))	− 2.47	0.1511

According to patients’ medical histories, 54% of patients had prior anthracycline exposure. Baseline Bazett’s QTc (QTcB) values for patients with prior anthracycline exposure were similar to those for patients with unknown or no prior anthracycline exposure. After CPX-351 treatment, there was no significant change in mean QTcB among patients with prior anthracycline exposure (Table 3), except 1 patient with prior anthracycline (doxorubicin) exposure > 450 mg/m^2^ who had QTcB measurements > 500 ms at baseline. Among patients without prior anthracycline exposure, there was no significant change in mean QTcB after CPX-351 treatment. QTcB data tabulated according to cumulative anthracycline exposure after CPX-351 treatment were compared to baseline QTcB values (Table 3). Cumulative exposure was calculated under the assumption that daunorubicin inside CPX-351 is the same as daunorubicin in conventional formulations. There were no significant changes in mean QTcB after CPX-351 treatment across all anthracycline exposure categories. For patients with cumulative anthracycline exposure of 600 to 749 mg/m^2^, mean QTcB was 444 and 432 ms at baseline and EOS, respectively.

**Table 3:** Comparison of QTcB values before and after CPX-351 treatment based on anthracycline exposure

Doxorubicin equivalent exposure^a^ (mg/m^2^)	*n*	QTcB (msec) at screening		QTcB (msec) at EOS	
		Mean	SD	Mean	SD
Prior anthracycline exposure					
< 100	4	431	16	437	21
100–199	6	420	27	428	20
200–299	0	–	–	–	–
300–399	2	443	0	472^b^	–
400–500	0	–	–	–	–
0 or unknown	14	433	19	433	18
Cumulative anthracycline exposure^c^					
100–199	12	432	18	432	19
200–299	5	436	22	445	18
300–399	6	428	30	422	8
400–500	3	432	20	444	40

QTcB was evaluated using a conventional 12-lead electrocardiogram instrument

*QTcB* Bazett's corrected QT interval, *EOS* end-of-study, *SD* standard deviation

^a^Anthracycline equivalence was calculated based on conversion factor of 1 doxorubicin = 1.5 daunorubicin, assuming daunorubicin inside CPX-351 is equivalent to daunorubicin in conventional formulation

^b^Only 1 subject provided an electrocardiogram read at the EOS

^c^Cumulative anthracycline exposure after CPX-351 treatment

### Pharmacokinetics of cytarabine and daunorubicin

**Fig. 2:**
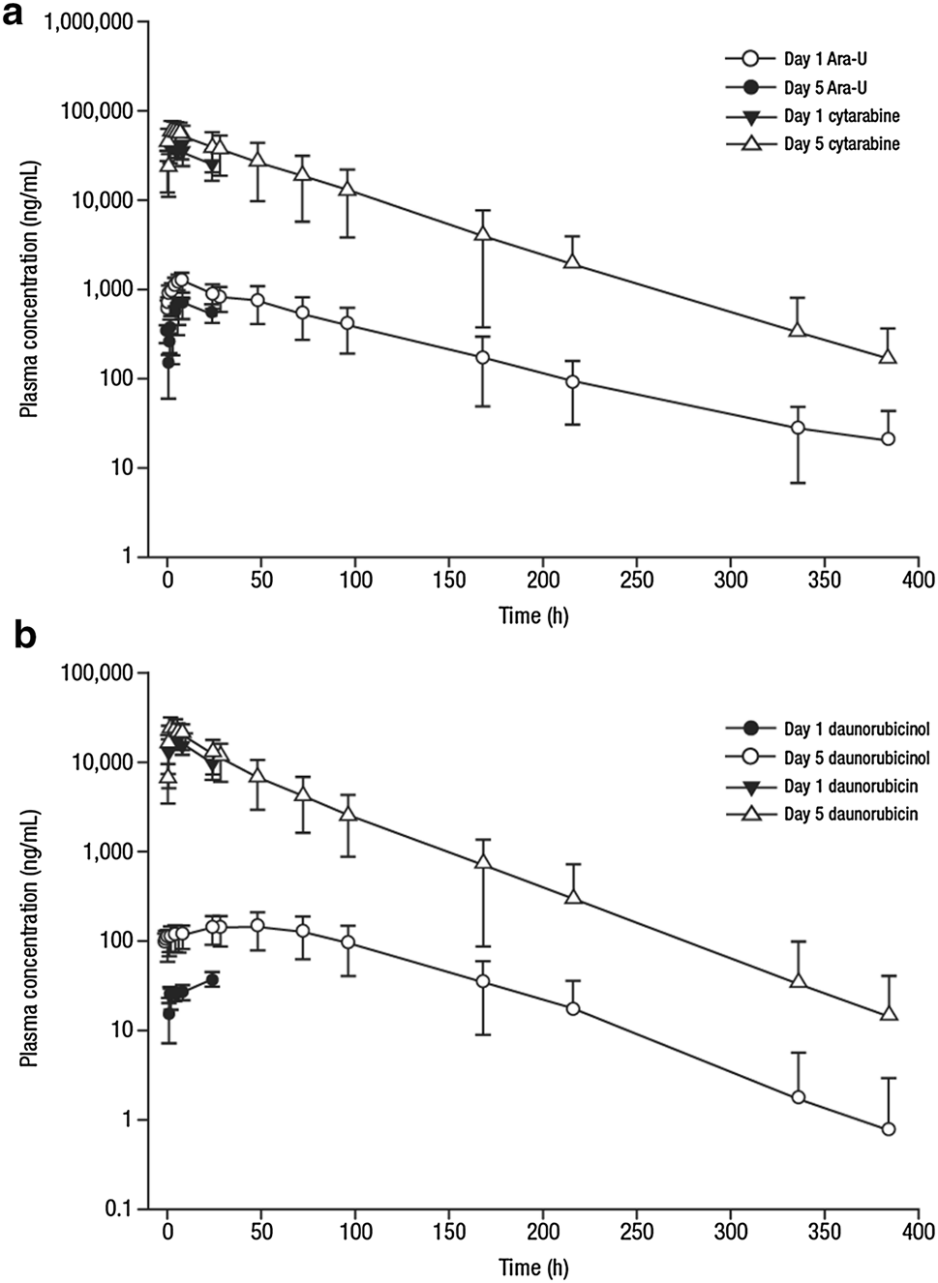
Plasma concentrations of total cytarabine (**a**) and daunorubicin (**b**). Pharmacokinetic samples from all patients (Groups A and B) were processed to determine concentrations of total plasma cytarabine and Ara-U (**a**) and daunorubicin and daunorubicinol (**b**)

Plasma pharmacokinetic parameters of total cytarabine and daunorubicin (and their respective metabolites, Ara-U and daunorubicinol) following Day 1 and Day 5 of CPX-351 administration are presented in Online Resource Table S3 and Table 4, respectively. Peak plasma concentrations of total cytarabine and total daunorubicin were reached near the end of the infusion and then declined in a mono-exponential manner (Fig. 2), thereby maintaining a synergistic ratio of cytarabine:daunorubicin in circulation for ≥ 48 h. The estimated mean (SD) *t*_1/2_ for total cytarabine and total daunorubicin was 40.4 (9.77) h and 31.5 (9.00) h, respectively. The estimated mean (SD) *t*_1/2_ for Ara-U and daunorubicinol was 60.3 (15.6) h and 43.9 (10.7) h, respectively, which were both slightly longer than those reported for their respective parent compounds. Overall, the mean (SD) metabolite:parent ratio for Ara-U and daunorubicinol was 3.22% (1.47%) and 1.79% (0.44%), respectively.

**Table 4:** Summary of pharmacokinetic parameters following Day 5 administration of CPX-351

	Cytarabine	Ara-U	Daunorubicin	Daunorubicinol
Mean *C*_max_ (SD), µg/mL	62.2 (20.9)	1.24 (0.25)	26.0 (8.5)	0.147 (0.52)
Median *T*_max_ (range), h	2.00 (0.75–8.33)	8 (5.78–8.33)	2.0 (0.75–6.02)	26.2 (1.5–48.0)
Mean AUC_0-48 h_ (SD), µg × h/mL	1900 (844)	44 (11.3)	637 (244)	6.24 (2.27)
Mean CLss (SD), L/h	0.131 (0.0791)		0.163 (0.087)	
Mean *V*_z_ (SD), L	7.11 (3.50)		6.64 (2.44)	
Mean *t*_1/2_ (SD), h	40.4 (9.77)	60.3 (15.6)	31.5 (9.0)	43.9 (10.7)
M:P ratio (%)		3.22 (1.47)		1.79 (0.443)
Mean cumulative dose recovered (SD), %	1.11 (0.35)	69.6 (33.9)	3.19 (0.91)	5.80 (2.49)
Mean CLr (SD), L/h	0.00167 (0.00102)	3.81 (2.02)	0.0061 (0.0028)	1.13 (0.74)
Total drug in urine,^a^ %	70.7 (33.8)^a^		9.00 (3.21)^a^	

*C*_*max*_ maximum observed concentration, *SD* standard deviation, *T*_*max*_ time of maximum observed concentration, *AUC* area under the concentration–time curve, *CLss* total body clearance, *Vz* volume of distribution based on the terminal elimination phase, *t*_*1/2*_ terminal phase half-life, *M:P ratio* metabolite to parent ratio, *CLr* renal clearance

^a^Percent of drug recovered in urine was the sum of parent and metabolite in urine during the 48-h interval following Day 5 administration of CPX-351

Over the 48-h dosing interval following Day 5 administration, the mean (SD) fraction of the dose recovered in urine as unchanged cytarabine was 1.11% (0.35%) and as Ara-U was 69.6% (33.9%); mean (SD) total urinary recovery of cytarabine plus Ara-U over the dosing interval was 70.7% (33.8%). The mean (SD) fraction of the dose recovered as unchanged daunorubicin was 3.19% (0.91%) and as daunorubicinol was 5.8% (2.49%); mean (SD) total urinary recovery of daunorubicin plus daunorubicinol was 9.0% (3.21%).

Copper pharmacokinetics were also monitored because the CPX-351 formulation contains copper. Copper concentrations peaked ~ 0.5 h after the end of infusion on Days 1 and 5; mean (SD) *C*_max_ was 963 (232) μg/dL on Day 1 and 1130 (306) μg/dL on Day 5, which was approximately 5 and 6 times higher than baseline (184 μg/dL), respectively. No AEs were attributed to elevated copper levels. Copper concentrations returned to baseline by a mean (SD) of 249 (100) h, corresponding to ~ 10.4 days, after the Day 5 dose.

### Efficacy

Overall, 13/26 (50%) patients responded to treatment (8 CR [31%] and 5 CRi [19%]). Eight of 13 (62%) patients with newly diagnosed AML responded, including 6 (46%) with CR and 2 (15%) with CRi. Four of 11 (36%) patients with relapsed/refractory AML responded, including 1 (9%) with CR and 3 (27%) with CRi. One of 2 (50%) patients with relapsed/refractory ALL responded with a CR. Twelve of 26 (46%) patients did not respond, and 1 patient with relapsed/refractory AML died in aplasia.

### Safety

All patients experienced AEs, but no patients discontinued study treatment due to an AE. One patient had grade 1 palpitations that delayed administration of CPX-351. The most frequently reported AEs were febrile neutropenia (73%), fatigue (54%), nausea (54%), decreased appetite (46%), and diarrhea (46%; Online Resource Table S4). Grade ≥ 3 AEs occurred in 85% of patients, most of which were grade 3. Five deaths were reported during follow-up, all attributed to progressive AML, including 2 (8%) patients who died before Day 60 (both with relapsed/refractory AML). No deaths were deemed related to CPX-351.

## Discussion

The 7 + 3 regimen has historically been restricted to patients with adequate cardiac function due to the risks associated with conventional cytarabine and daunorubicin formulations. Anthracyclines, such as daunorubicin, have been associated with cardiotoxicity, including left ventricular dysfunction and QT prolongation, both contemporaneously with administration and years after treatment [17, 18, 21, 22]. Although earlier reports suggested no correlation between acute and chronic cardiotoxicity, more recent research found that acute cardiotoxicity, such as QT prolongation, can be used to gauge anthracycline-induced cardiac damage [26, 27]. QTc prolongation was reported after anthracycline treatment in patients with newly diagnosed acute leukemia and correlated with left ventricular dysfunction on echocardiography [20]. QTc prolongation has also been reported following anthracycline treatment of other malignancies [19, 28].

Anthracycline-induced QT prolongation is not just an acute and reversible phenomenon, as QT prolongation has been observed in cancer survivors long after treatment [28, 19]. Therefore, QT interval length has been proposed as a biomarker of anthracycline-induced cardiotoxicity [20, 26]. Although the mechanism underlying anthracycline-induced cardiotoxicity is not completely understood, myocardial damage has been detected after a single anthracycline dose in animal models [26]. Acute damage to cardiac tissues may result in cardiomyocyte apoptosis, leakage of cardiac enzymes into the circulation, and conduction disturbances [26]. Damage may not manifest as clinical symptoms, however, until myocardial reserves are depleted and compensation is no longer possible [27]. QTc measurement enables detection of damage before changes in measures of cardiac function, such as LVEF [26]. QTc prolongation correlated with left ventricular dysfunction [20]; however, like with other noninvasive biomarkers, the utility of QTc prolongation in predicting heart failure in cancer survivors has not yet been established [19, 20, 27–29].

In contrast to historical evidence with 7 + 3, the current study found no clinically meaningful effects of CPX-351 on cardiac repolarization, heart rate, PR, or QRS. On Day 1, the upper limit of change in QTcF rarely exceeded 10 ms, and changes that did were not statistically significant. Concentration–response analyses did not show an increase in QTcF with increasing exposure to any component or metabolite of CPX-351. This does not mean patients treated with CPX-351 may not experience QT prolongation, as a recent meta-analysis of QT prolongation following anthracycline treatment found QT prolongation was often associated with common supportive-care drugs [16]. However, it is unlikely CPX-351 would independently contribute to QT prolongation. Unlike prior studies that prohibited QT prolongation agents, the use of certain QT prolongation agents (eg, ondansetron) was permitted in this study. Even in the presence of these medications, no significant QTc change following CPX-351 treatment was observed acutely or sub-chronically, and CPX-351 caused no significant reductions in LVEF, even at the highest cumulative dose (data not presented). However, since the study limited the use and types of supportive care, these results may not be generalizable to individuals receiving supportive care medicines known to induce QT prolongation [16].

In the current study, neither mean QTcB nor the frequency of prolonged QTcB > 450 ms significantly increased following CPX-351 treatment, regardless of prior anthracycline exposure, except 1 patient with prior anthracycline exposure > 450 mg/m^2^. QTc observation in this patient was complicated by concurrent use of QTc prolongation medications, such as ondansetron, pantoprazole, and palonosetron. Prior anthracycline exposure was previously identified as the most consistent risk factor for cardiotoxicity and may predispose patients to develop cardiotoxicity below known thresholds of respective anthracyclines [27]. Although QTc is only 1 marker of cardiac injury, these encouraging results suggest that CPX-351 induces minimal myocardiocyte damage. In this study, cumulative anthracycline dose was calculated using an estimated 1:1 equivalence ratio for daunorubicin in CPX-351 to conventional daunorubicin. However, this equivalence ratio may be too conservative and preclude patients from receiving CPX-351 treatment. More studies are needed to determine a more accurate CPX-351 anthracycline conversion factor and maximum CPX-351 equivalent anthracycline exposure.

The lack of QT interval prolongation observed with CPX-351 may be partly attributed to its pharmacokinetic profile. Consistent with the phase 1 study [30], the small volume of distribution suggested limited distribution outside the blood compartment and limited interaction with off-target tissues. In prior analyses, the majority of CPX-351 remained encapsulated during the first 24 h post-SOI [30, 31]. Studies of single-agent daunorubicin liposomes reported stable drug encapsulation and reduced cardiotoxicity compared with free daunorubicin [17, 18, 21, 22]. The retention of daunorubicin and cytarabine in CPX-351 liposomes may provide similar cardioprotection.

Encapsulation of cytarabine and daunorubicin in CPX-351 liposomes provides significant pharmacokinetic and pharmacological advantages over conventional formulation, as previously discussed [30]. The half-lives of cytarabine and daunorubicin after CPX-351 administration were prolonged compared with those previously reported after administration of free drug [7, 8]. Approximately, 50% of the administered dose of CPX-351 remained in circulation > 30 h after injection; in contrast, > 90% of daunorubicin distributed rapidly after injection of free drug, and the 18.5-h half-life of free daunorubicin reported in the literature reflected the terminal half-life of the remaining < 10% [8]. Similar to prior analyses, CPX-351 maintained a synergistic ratio of cytarabine:daunorubicin for up to 48 h after administration [30]. Due to bioanalytical issues, free drug concentrations were not successfully determined and are not reported here.

The cytarabine and daunorubicin in CPX-351 liposomes followed the same metabolism and elimination pathways as free drugs. Following CPX-351 administration, the majority of cytarabine, but not daunorubicin, was subjected to renal elimination. Overall, the percent of total cytarabine and daunorubicin and their respective metabolites eliminated in urine was consistent with previous reports [32, 33]. However, the renal elimination rates for daunorubicin and cytarabine following CPX-351 treatment were much slower than those reported following treatment with free drugs.

In this phase 2 study, the efficacy and safety profile of CPX-351 were consistent with previous clinical trials. Here, an overall remission rate of 50% was observed, which is similar to the 48% and 49% reported in the phase 3 study of adults with newly diagnosed high-risk/secondary AML and the phase 2 study of adults with relapsed/refractory AML, respectively [12, 34, 35]. The overall safety profile of CPX-351 was consistent with prior clinical studies and the known safety profile of 7 + 3 [11, 12, 34, 35]. No copper-related events were observed, suggesting that most copper remained encapsulated or otherwise associated with the liposomal membrane, reducing its bioavailability. Alternately, the transient elevation of copper may not have allowed sufficient exposure to produce symptoms. No treatment-related discontinuations occurred and all deaths were attributed to progressive disease.

In summary, cytarabine and daunorubicin encapsulated in CPX-351 liposomes were metabolized and excreted similarly to their respective conventional formulations. CPX-351 did not prolong the QT/QTc interval acutely or sub-chronically, suggesting CPX-351 may cause less cardiotoxicity than previously reported for conventional daunorubicin.

## Electronic supplementary material

Below is the link to the electronic supplementary material.

Supplementary file1 (DOCX 36 kb)
