# Supplementary material for: A phase 2 study to assess the pharmacokinetics and pharmacodynamics of CPX-351 and its effects on cardiac repolarization in patients with acute leukemias
Source: Cancer Chemother Pharmacol. 2019 May 16;84(1):163–73. doi: 10.1007/s00280-019-03856-9 (PMC6562048; doi:10.1007/s00280-019-03856-9)
Supplement: Supplementary file 1 — Supplementary file1 (DOCX 36 kb) [file 280_2019_3856_MOESM1_ESM.docx]

**Online Resource**

**Table S1. Anthracyclines Equivalent Guidelines^a^**

| **Drug** | **Conversion factor^b^** |
| --- | --- |
| Daunorubicin | 1 |
| Doxorubicin | 2 |
| Epirubicin | 1 |
| Idarubicin | 4 |
| Mitoxantrone | 4.4 |

^a^Adapted from Keefe D, et al. Anthracycline-induced cardiomyopathy. Seminars in Oncology. 2001;28(4 Suppl 12):2-7.

^b^To calculate the equivalent dose of daunorubicin, the total cumulative dose of anthracycline was multiplied by the conversion factor.

**Table S2. Patient Disposition**

|  | **Patients receiving CPX-351 N = 26** |
| --- | --- |
| Terminated treatment, n (%) | 25 |
| Persistent disease | 8 (31) |
| Received HCT | 7 (27) |
| Administered non-protocol chemotherapy | 3 (12) |
| Investigator discretion | 3 (12) |
| Consent withdrawn | 2 (8) |
| Intercurrent illness | 1 (4) |
| Relapsed disease | 1 (4) |
| Completed treatment | 1 (4) |

HCT, hematopoietic cell transplant.

**Table S3. Pharmacokinetic Parameters for Cytarabine and Daunorubicin Following Day 1 of CPX-351 Administration**

|  | **Cytarabine** | **Ara-U** | **Daunorubicin** | **Daunorubicinol** |
| --- | --- | --- | --- | --- |
| Mean C_max_ (SD), µg/mL | 46.0 (12.6) | 0.71 (0.21) | 23.1 (6.6) | 0.0366 (0.0062) |
| Median T_max_ (range), h | 2.01 (0.78, 6.07) | 8.03 (7.93, 24.03) | 2.00 (0.78, 4.37) | 24.0 (0.78, 24.12) |
| Mean AUC_0-24h_ (SD), µg*h/mL | 765 (216) | 13.5 (3.5) | 340 (87.9) | 0.69 (0.11) |

C_max,_ maximum observed concentration; SD, standard deviation; T_max_, time of maximum observed concentration; AUC_,_ area under the concentration-time curve.

**Table S4. Summary of AEs Occurring in ≥20% of Patients**

| **Preferred term** | **N = 26** |
| --- | --- |
| Any AE, n (%) | 26 (100) |
| AEs reported in >20% of patients, n (%) |  |
| Febrile neutropenia | 19 (73) |
| Fatigue | 14 (54) |
| Nausea | 14 (54) |
| Decreased appetite | 12 (46) |
| Diarrhea | 12 (46) |
| Constipation | 11 (42) |
| Headache | 11 (42) |
| Insomnia | 11 (42) |
| Peripheral edema | 10 (38) |
| Vomiting | 10 (38) |
| Cough | 9 (35) |
| Hypotension | 8 (31) |
| Chills | 7 (27) |
| Hypoxia | 7 (27) |
| Oropharyngeal pain | 7 (27) |
| Abdominal pain | 6 (23) |
| Arthralgia | 6 (23) |
| Back pain | 6 (23) |
| Confusional state | 6 (23) |
| Epistaxis | 6 (23) |
| Pyrexia | 6 (23) |
| AEs of special interest, n (%) |  |
| Infection AEs | 21 (81) |
| Bleeding AEs | 23 (88) |
| Cardiac AEs | 12 (46) |
| Grade 3 AEs, n (%) | 17 (65) |
| Grade 4 AEs, n (%) | 3 (12) |
| Grade 5 AEs,^a^ n (%) | 2 (8) |
| Serious AEs, n (%) | 8 (31) |
| Discontinuations due to AEs, n (%) | 0 (0) |

AE, adverse event.

^a^Deaths before Day 60; both were attributed to progressive AML and occurred in patients with relapsed/refractory AML.
